# Exploring the Role of the MAPK Signalling Pathway in Dog Immunity: From Cancer to Parasitic Diseases

**DOI:** 10.1111/pim.70083

**Published:** 2026-05-18

**Authors:** Francini Neves Ribeiro, Fernanda Nazaré Morgado, Renato Porrozzi

**Affiliations:** ^1^ Laboratório de Protozoologia Instituto Oswaldo Cruz, Fundação Oswaldo Cruz, FIOCRUZ Rio de Janeiro Brazil; ^2^ Laboratório de Imunoparasitologia Instituto Oswaldo Cruz, Fundação Oswaldo Cruz, FIOCRUZ Rio de Janeiro Brazil; ^3^ Departamento de Fisiopatologia Escola de Ciencias da Saúde e Bem‐Estar, Faculdades Souza Marques Rio de Janeiro Brazil

**Keywords:** cancer, cell signalling, dogs, immune system, MAPK pathway, parasite survival, parasitic disease

## Abstract

Mitogen‐activated protein kinases (MAPKs) are intracellular signalling proteins that modulate several cellular processes, such as proliferation, cytokine production and survival. Like humans, dogs may exhibit changes in MAPK signalling under pathological conditions. Alterations in this pathway have been observed in several types of cancers in dogs and have been widely studied in veterinary oncology. In humans, several studies have focused on the MAPK pathway in cancer, inflammatory diseases and parasitic diseases; however, the role of the MAPK pathway in parasitic diseases has not been extensively explored in canine models. Some parasites use these proteins as strategic targets for modulating the immune response to ensure their survival and persistence in host cells. Although they have distinct pathogeneses, some mechanisms of immune system evasion are shared between parasites and cancer cells. The aim of this review is to discuss the MAPK pathway involvement in the immune system and in inflammatory and parasitic disease in dogs.

## 1 Introduction

In cell biology, the activation of several mechanisms that mediate intracellular signalling is necessary for a cell to respond to external stimuli and produce a final response. Several intracellular signalling proteins and transcription factors are involved in this process that together modulate the cell response and cell fate. Among them are the mitogen‐activated protein kinases (MAPKs) [1]. MAPKs are part of the serine (Ser)/threonine (Thr) family of protein kinases that play key roles in the modulation of several intracellular mechanisms. MAPK activation occurs through a phosphorylation cascade involving MAP3Ks, MAP2Ks and MAPKs. In some cases, MAP4Ks contribute to MAP3Ks activation [1, 2]. In terms of their activation, these kinases can be divided into extracellular signal‐regulated protein kinases (ERKs), p38 MAPK, c‐Jun N‐terminal kinases (JNKs) and ERK5 [1] (Table 1). MAPKs are present in all eukaryotes and can be activated by a range of extracellular stimuli [3], playing key roles in cell proliferation, survival, motility, apoptosis and stress responses [1]. Owing to its central role in cell biology, MAPK dysregulation is involved in several diseases, such as cancer and autoimmune diseases [1]. Like humans, dogs exhibit changes in this signalling pathway under pathological conditions. In dogs, changes in MAPK signalling have been observed in several types of cancer [4, 5, 6], autoimmune diseases [7] and inflammatory processes [8, 9, 10]; however, its dysregulation in parasitic diseases has not been extensively explored in a canine model. These proteins are strategic targets of parasites to subvert the immune response, ensuring their survival and persistence in intracellular niches [11]. Although they have distinct pathogeneses, some characteristics of immune evasion are shared between cancer and parasitic diseases. Several mechanisms used by parasites to interact with and subvert the host immune response have been observed in cancer cells [12]. Thus, this review focuses on how MAPK signalling modulates the immune response in dogs, where we address the participation and dysregulation of this protein in inflammatory processes and cancer and its modulation by intracellular protozoa. Data obtained from murine model and humans are also included for comparison.

**TABLE 1:** Expression/Effector mechanisms of the MAPK pathway in inflammatory processes, cancer, and parasitic diseases in dogs.

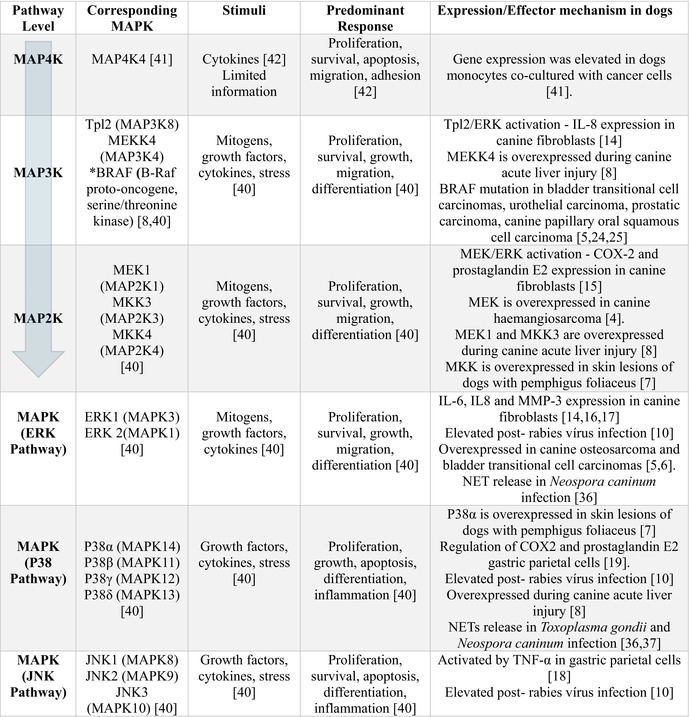

*Note:* The Mitogen‐activated protein kinase (MAPK) mediate extracellular stimuli into effector responses. This pathway is activated through the sequential phosphorylation of MAP4K, MAP3K, MAP2K, and MAPK (arrow), resulting in the activation of three classical MAPKs: ERK (Extracellular Signal‐Regulated Kinase), P38 MAPK, and JNK (c‐Jun N‐terminal kinase), which in turn promote the activation of various effector mechanisms in canine cells. *BRAF (B‐Raf proto‐oncogene, serine/threonine kinase) gene mutations results in constitutive activation of MEK‐ERK pathway, independent of extracellular stimuli. MEK1 (Mitogen‐Activated Protein Kinase Kinase 1), MKK (MAP Kinase Kinase), MKK3 (Mitogen‐activated protein kinase kinase 3), MEKK4 (MAP kinase kinase kinase 4), Tpl2 (Tumor progression locus 2), NETs (Neutrophil Extracellular Traps).

## 2 MAPKs in the Immune Response and Inflammation in Dogs

MAPKs play a key role in the immune response signalling for induction of several cellular activities toward an effector mechanism [13]. In dogs, the MAPK pathway is associated with several inflammatory processes [7, 8, 9]. In skin diseases such as atopic dermatitis and pemphigus foliaceus, the MAPK is essential in modulating the immune response and cytokine production [7, 14] Dermal fibroblasts contribute significantly to the production of a broad spectrum of cytokines involved in the pathogenesis of skin diseases [14]. In this context, IL‐1β is an important cytokine that promotes activation of the MAPK signalling pathway and induces the expression of various cytokines in a range of cell types, including dermal fibroblasts [14, 15]. The IL‐1β‐stimulated IL‐8 expression in canine fibroblasts is mediated by ERK1/2 activation. In this pathway, the MAP3K Tpl2 acts as a regulator of ERK1/2 upstream signalling [14]. In addition, ERK plays key roles in inducing the expression of IL‐6 and matrix metalloproteinases (such as MMP‐3) mediated by IL‐1β in canine fibroblasts [16, 17]. This activation results in phosphorylation of ERK1/2 but not in the JNK or p38 MAPK pathways [16, 17]. Therefore, ERK1/2 appears to be predominantly involved in the modulation of this response [14, 16, 17]. In addition to their key role in cytokine expression, MAPKs are essential for the expression of inflammatory mediators such as cyclooxygenase 2 (COX‐2) and the subsequent synthesis of prostaglandin E2 [15]. The activation of ERK and its upstream MEK pathway is essential for COX‐2 expression and IL‐1β‐induced prostaglandin E2 synthesis in canine dermal fibroblasts. In addition to the MAPK pathway, NF‐κB contributes significantly to IL‐1β‐induced COX‐2 expression. The inhibition of NF‐κB inhibited IL‐1β‐induced MEK/ERK activation, indicating that NF‐κB plays an important role in IL‐1β‐induced ERK activation, as well as in COX‐2 expression and prostaglandin E2 release in canine fibroblasts [15]. Thus, the MAPK pathway can be activated in association with other signalling pathways. In the skin lesions of dogs with pemphigus foliaceus (PF), MAPK proteins are overexpressed. In this context, p38 MAPK pathway genes, including MAPK14 and MAP2K4 are upregulated [7]. In addition to the MAPK pathway, JAK/STAT and NF‐κB were also upregulated in the skin lesions of dogs with PF, indicating the dysregulation and crosstalk of intracellular signalling pathways in the dog inflammatory response [7]. Not only in skin inflammatory conditions is the MAPK pathway dysregulated, but also in gastric and hepatic inflammatory processes [8, 18, 19]. In gastric parietal cells, the expression of COX2 and prostaglandin E2 is regulated by p38 MAPK and NF‐κB [19]. Additionally, TNF‐α plays a key role in the activation of the JNK pathway [18]. Studies by Tao et al. reported that, compared with those in control dogs, several MAPKs—such as MAP3K4 (MEKK4), MAP2K3 (MKK3), p38 MAPK and MAP2K1 (MEK1)—were overexpressed during canine acute liver injury, indicating that this pathway plays a strong role in the hepatic inflammatory process [8]. Dogs and humans share several similarities in their inflammatory responses. The inflammatory response induced by the rabies virus in dogs, which results in encephalomyelitis in mammals, reflects the inflammatory process observed in human infection. MAPKs are highly expressed in this context, contributing to the expression of various chemokines and cytokines in response to viral infection. In neuronal cells from dogs and humans, phosphorylation of p38, ERK and JNK is elevated post‐infection with the rabies virus. This virus‐induced activation of MAPKs can activate other signalling pathways, such as NF‐κB. In this context, NF‐κB activation in brain tissues is indirectly mediated by the MAPK pathway following infection. In comparison with dogs and humans, lower levels of MAPK and NF‐κB pathway activation are observed in brain tissues from artificially infected mice, as well as lower levels of cytokines and chemokines. Therefore, the similarity of this response between dogs and humans indicates that the canine model more accurately reflects the pathogenesis observed in humans compared to the murine model of infection [10].

## 3 MAPKs in Cancer

**FIGURE 1:**
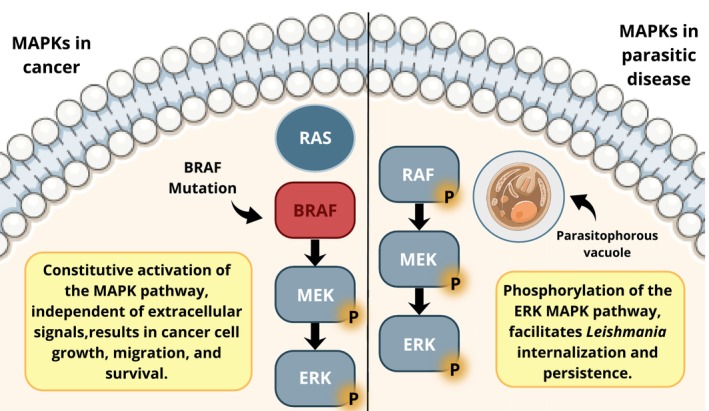
Representative scheme about the role of MAPKs in cancer cells and *Leishmania*‐infected cells. In dogs' cancer cells, mutation of the BRAF gene results in constitutive phosphorylation of this MAPK pathway, causing proliferation and survival of these cells (left). During *Leishmania* infection in mice, there is phosphorylation of the RAF–MEK–ERK pathway, contributing to the internalization and persistence of the parasite in the host cell (right). RAS, rat sarcoma virus; BRAF, B‐Raf proto‐oncogene, serine/threonine kinase; RAF, rapidly accelerated fibrosarcoma; MEK, mitogen‐activated protein kinase kinase; ERK, extracellular signal‐regulated kinase.

Overactivation of MAPK signalling results in the survival and proliferation of cancer cells [20]. In humans, the RAS–RAF–MEK–ERK pathway is altered in several types of cancer. These alterations occur primarily owing to mutations in BRAF (B‐Raf Proto‐Oncogene, serine/threonine kinase) and its upstream activator RAS [21]. RAS proteins are small guanosine triphosphate (GTP)‐binding proteins (GTPases) that transduce signals from membrane receptors to intracellular signalling pathways. The RAS protein promotes the recruitment and phosphorylation of RAF, which in turn phosphorylates MEK (MAP2K), which then phosphorylates ERK (MAPK) [22]. Hyperactivation of this pathway is strongly involved in cancer in humans [22], resulting in the dysregulation of cell proliferation, resistance to apoptosis and metastasis [23]. Therefore, several molecules have been developed for the targeted inhibition of this pathway [22]. Mutation of the BRAF gene results in constitutive activation of MAPK signalling, resulting in the proliferation of cancer cells independent of extracellular stimuli [24] (Figure 1). Several types of cancer cells in dogs have BRAF mutations, resulting in dysregulation of the MAPK pathway and contributing to the pathogenesis of canine cancer [24, 25, 26]. The RAS–RAF–MEK–ERK pathway is involved in most canine oral tumours of epithelial origin [27]. Compared with that in healthy animals, MEK (MAP2K) is overexpressed in dogs with haemangiosarcoma [4]. In addition, mutations in the BRAF gene and constitutive phosphorylation of ERK1/2 have been detected in bladder transitional cell carcinomas [5]. Thus, the BRAF/MAPK pathway can also be considered a potential therapeutic target in veterinary oncology [24, 25]. Canine BRAF has high homology with human BRAF [5]. In a comparative study between canine and human mucosal melanoma, Simpson et al. (2014) considered dogs as preclinical models for human disease because humans and dogs share significant clinical and histopathological characteristics [28]. In this context, both human and canine melanoma cells exhibit similar expression patterns in the MAPK pathway [29]. In canine and human osteosarcoma cancer cells, the overexpression of EphA2 receptor was observed. This overexpression in both species mediates pro‐oncogenic signalling through the activation of the AKT, ERK1/2 and SRC pathways, promoting the proliferation and viability of cancer cells [6]. Therefore, due to the similarity in molecular and immunological characteristics between dogs and humans, the translational strategy for studying cancer pathogenesis and therapeutic, human and veterinary medicine complement each other, increasing the knowledge of pathogenesis in benefits of both species [30].

## 4 MAPKs in Parasitic Diseases

Several studies have been conducted in murine models to evaluate the roles of MAPKs in parasitic diseases. Host–pathogen interactions can modulate several intracellular signalling pathways to subvert the immune system and promote parasite survival and persistence [31]. Parasites of the genera *Leishmania* and *Trypanosoma*, which cause Leishmaniasis and Chagas disease, respectively, can modulate the p38 MAPK and ERK1/2 pathways of macrophages and dendritic cells, ensuring their survival in the intracellular niches of host cells [11]. The activation of the RAF–MEK–ERK pathway plays a key role in the internalization of *Leishmania* by macrophages and in the pathogenesis of leishmaniasis (Figure 1). *Leishmania* amastigotes can phosphorylate RAF‐1, MEK and ERK in macrophages after they are phagocytosed, and inhibition of this pathway reduces their survival in intracellular niches [31]. Among the various mechanisms involved in the modulation of MAPK activity by *Leishmania*, the regulation of phosphatases plays important roles modulating the immune system. A study by Srivastava et al. revealed increased MPK‐1 phosphatase activity and the downregulation of MPK‐3 by *Leishmania major*. MPK‐1 is involved in the dephosphorylation of p38 MAPK (IL‐12 and NO production) [32]. On the other hand, MPK‐3 is involved in the dephosphorylation of ERK 1/2 (IL‐10 production). As a survival strategy, *L. major* modulates phosphatases by diverting intracellular signals to ERK1/2 activation and IL‐10 production, reducing p38 MAPK activation and IL‐12 production [32]. *Leishmania amazonensis* was also shown to modulate the MAPK pathway to ensure its persistence after infection. In this study, the *Leishmania* parasite prevented the proper maturation of dendritic cells. This occurred through the activation of ERK1/2 [33]. Although the roles of MAPKs in the modulation of the immune response in canine parasitic diseases has not been well explored, Melo et al. [34] evaluated the expression of p38 MAPK in the macrophages of dogs infected with *Leishmania infantum*. Unlike macrophages from healthy dogs, macrophages from dogs infected with *L. infantum* cannot significantly increase p38 MAPK phosphorylation after stimulation with lipopolysaccharide (LPS) [34], demonstrating that the alteration of this pathway is probably induced by *Leishmania* infection in dogs. In addition, the infection with *L. infantum* can modulate the early immune response through the upregulation of several microRNAs in dog cells [35]. The MAPK pathway is one of the intracellular signalling pathways most affected by upregulated miRNAs during *Leishmania* infection in canine peripheral blood mononuclear cells. Thus, the MAPK signalling pathway is considered an important cellular signalling pathway subverted by *Leishmania* and could be modulated by upregulated miRNAs [35]. In this context, the MAPK pathway is important for host–pathogen interactions and seems to play an important role in *Leishmania* infection in dogs. However, further studies should be conducted to elucidate the role of the MAPK pathway in the modulation of the canine immune response. In addition to those involved in leishmaniasis, canine MAPKs are involved in the modulation of the immune response in other parasitic diseases, such as toxoplasmosis and neosporosis [36, 37]. The inhibition of P38 MAPK in dog neutrophils stimulated with *Toxoplasma gondii* tachyzoites significantly inhibited the Neutrophil Extracellular Traps (NETs) release, while the inhibition of the p38 and ERK1/2 pathways in dog neutrophils stimulated with *Neospora caninum* tachyzoites also significantly inhibited NET release, demonstrating that this process depends on the activation of the MAPK signalling pathway in dog cells stimulated by these parasites [36, 37].

## 5 MAPKs As Therapeutic Targets

Considering the multiple roles of MAPKs in cellular processes, some limitations and possible side effects should be considered when considering this pathway as a potential therapeutic target [38]. In canine models, the MAPK pathway has been the target of therapies to treat inflammatory processes [15] and cancer [4, 27]. MEK inhibitors have been widely studied as targeted therapies for the treatment of neoplasms in veterinary oncology [27, 38]. A clinical study conducted by Takada et al. showed that a MEK inhibitor was safe and well tolerated by dogs with cancer [38]. The MEK pathway is also important in the pathogenesis of parasitic diseases, such as leishmaniasis [31]. Studies by Barrie et al., showed that the use of a MEK inhibitor used in cancer treatment reduced the parasite load and also pathogenesis of mice infected with *Leishmania*, indicating the ERK/MAPK pathway as a potential therapeutic target in leishmaniasis [31]. In addition, Zhao et al. proposed the MAPK signalling pathway as an interesting target for the diagnosis, prevention and potential treatment of parasitic diseases [39]. Therefore, a better understanding of the molecules involved in the parasite–host relationship can help identify target molecules for treatments aimed at reducing parasite survival and persistence in host cells [12].

## 6 Conclusion

Several studies have been conducted to better understand the role of MAPKs in parasitic diseases and their potential as therapeutic targets. However, most studies investigating the role of MAPKs in dogs have focused on cancer. Some studies have shown changes in the canine MAPK pathway, emphasizing the relevance of this pathway in dogs. However, its role in parasitic diseases should be further explored in canine models to better understand host–pathogen interactions and identify potential therapeutic targets within this pathway and the possible associated side effects.

## Author Contributions

The authors contributed equally to the realisation of this work.

## Funding

This work was supported by the Fundação Carlos Chagas Filho de Amparo à Pesquisa do Estado do Rio de Janeiro: E‐26/211.340/2021 : E‐26/204.308/2024. Conselho Nacional de Desenvolvimento Científico e Tecnológico ‐ PROEP ‐ IOC: 441708/2024 : 442053/2024‐0. Coordenação de Aperfeiçoamento de Pessoal de Nível Superior ‐ Brasil (CAPES) ‐ Finance code: 001.

## Conflicts of Interest

The authors declare no conflicts of interest.

## Data Availability

Data sharing not applicable to this article as no datasets were generated or analysed during the current study.
